# Supplementary material for: Identification of photoperiod-induced specific miRNAs in the adrenal glands of Sunite sheep (Ovis aries)
Source: Front Vet Sci. 2022 Jul 22;9:888207. doi: 10.3389/fvets.2022.888207 (PMC9354845; doi:10.3389/fvets.2022.888207)
Supplement: Supplementary file 1 [file Data_Sheet_1.ZIP › Supplementary file/miRDeep2 file.zip!/miRDeep2 file/Know_Structure/SP42A2/oar-mir-30c.pdf]

miRBase precursor : oar-mir-30c
Total read count : 108428
oar-miR-30c read count : 108256
remaining reads : 172

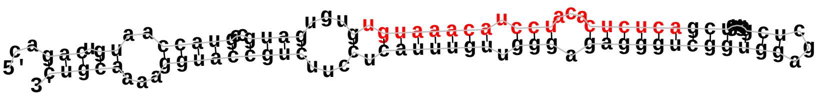

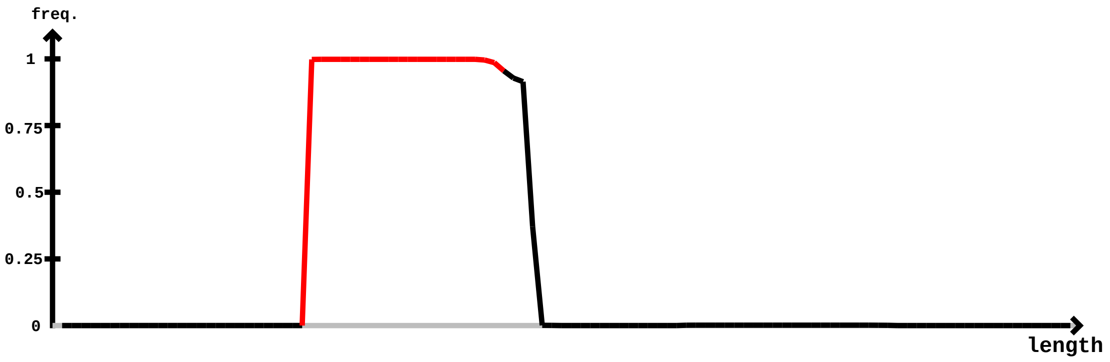


```
                                  oar-miR-30c
5'- cagacuguaaccaugccguagugugUguaaacauccuacacucucagcugugagcucgagguggcugggagaggguuguuuacuccuucugccauggaaaacguc   -3'    exp
    ..(((.((..(((((..((((...(.(((((((.(((...((((((((....(((...))))))))))).))).))))))).)...)))))))))...)))))    reads      mm        sample
    cagacuguaaccaugccguagugug................................................................................    1          0         seq
    .agacuguaaccaugccguagugug................................................................................    1          0         seq
    ....cuguaaccaugccguagugug................................................................................    3          0         seq
    .....uguaaccaugccguagugug................................................................................    1          0         seq
    ........................guguaaacauccuacacucucagc.........................................................    1          0         seq
    .........................uguaaacauccuUcacu...............................................................    1          1         seq
    .........................uguaaacauccuacacu...............................................................    4          0         seq
    .........................uguaaacauccuacacuc..............................................................    217        0         seq
    .........................uguaaacaCccuacacuc..............................................................    3          1         seq
    .........................uguaaacauccuacacuU..............................................................    64         1         seq
    .........................uguaaacauccuacacucu.............................................................    962        0         seq
    .........................uguaaacauccuacacuUu.............................................................    3          1         seq
    .........................uguGaacauccuacacucu.............................................................    5          1         seq
    .........................uguaaacaCccuacacucu.............................................................    84         1         seq
    .........................uguaaacauccuacacucuc............................................................    3095       0         seq
    .........................uguaaacaCccuacacucuc............................................................    269        1         seq
    .........................uguaaacaCccuacacucuca...........................................................    257        1         seq
    .........................uguaaacauccuacacucuca...........................................................    2651       0         seq
    .........................uguaaacaCccuacacucucag..........................................................    177        1         seq
    .........................uguaaacauccuacacucucag..........................................................    1307       0         seq
    .........................uguaaacaCccuacacucucagc.........................................................    5144       1         seq
    .........................uguaaacauccuacacucucagc.........................................................    53792      0         seq
    .........................uguaaacauccuacacucucagcu........................................................    36907      0         seq
    .........................uguaaacaCccuacacucucagcu........................................................    3158       1         seq
    .........................uguaaacauccuacacucucagcug.......................................................    32         0         seq
    .........................uguaaacaCccuacacucucagcug.......................................................    2          1         seq
    .........................uguaaacauccuacacucucagcugu......................................................    65         0         seq
    .........................uguaaacaCccuacacucucagcugu......................................................    2          1         seq
    ..........................guaaacauccuacacuc..............................................................    2          0         seq
    ..........................guaaacauccuacacucu.............................................................    2          0         seq
    ..........................guaaacauccuacacucuc............................................................    1          0         seq
    ..........................guaaacauccuacacucuca...........................................................    2          0         seq
    ..........................guaaacaCccuacacucucagc.........................................................    3          1         seq
    ..........................guaaacauccuacacucucagc.........................................................    22         0         seq
    ..........................guaaacaCccuacacucucagcu........................................................    1          1         seq
```

oar-miR-30c

cagacuguaaccaugccguagugugu**uguaaacauccuacacucuca**gcugugagcucgagguggcugggagaggguuguuuacuccuucugccauggaaaacguc

```
cagacuguaaccaugccguaguguguguaaacauccuacacucucagcugugagcucgagguggcugggagaggguuguuuacuccuucugccauggaaaacguc
..........................guaaacauccuacacucucagcu........................................................ 16 0 seq
...........................uaaacauccuacacucucagcu........................................................ 1 0 seq
............................aaacauccuacacucucagc......................................................... 1 0 seq
............................aaacaCccuacacucucagc......................................................... 1 1 seq
...............................cauccuacacucucagc......................................................... 1 0 seq
................................auccuacacucucagcu........................................................ 1 0 seq
................................................................cugggagaggguuguuuacu..................... 31 0 seq
................................................................cugggagaggguuguuuacuc.................... 27 0 seq
................................................................cugggagaggguuguuuacucc................... 83 0 seq
................................................................cugggagaggguuguuuacuccu.................. 2 0 seq
................................................................cugggagaggguuguuuacuccuu................. 2 0 seq
.................................................................ugggagaggguuguuuacu..................... 6 0 seq
.................................................................ugggagaggguuguuuacuc.................... 1 0 seq
.................................................................ugggagaggguuguuuacucc................... 13 0 seq
.................................................................ugggagaggguuguuuacuccu.................. 1 0 seq
```
